# Cross-cultural validation of the Weight Stigma Exposure Inventory (WeSEI): secondary data analysis from Taiwan, China, Hong Kong, Indonesia, Türkiye, and Malaysia

**DOI:** 10.1186/s40337-025-01480-y

**Published:** 2025-12-13

**Authors:** Chia-Wei Fan, Kamolthip Ruckwongpatr, I-Ching Lin, I-Hua Chen, Ji-Kang Chen, Ira Nurmala, Muthmainnah Muthmainnah, Wan Ying Gan, Servet Üztemur, Yen-Ling Chang, Chien-Chin Lin, Jeanette Reffstrup Christensen, Nadia Bevan, Mark D. Griffiths, Amir H. Pakpour, Chung-Ying Lin

**Affiliations:** 1 Department of Occupational Therapy, AdventHealth University, Orlando, FL USA; 2 Department of Physical Therapy, College of Health Sciences, Christian University of Thailand, Nakhon Pathom, Thailand; 3 Department of Healthcare Administration, Asia University, Taichung, Taiwan; 4 Department of Family Medicine, Asia University Hospital, Taichung, Taiwan; 5 Department of Kinesiology, Health, and Leisure, Chienkuo Technology University, Changhua, Taiwan; 6 Chinese Academy of Education Big Data, Faculty of Education, Qufu Normal University, Qufu, China; 7 Department of Social Work, Chinese University of Hong Kong, Shatin, New Territories Hong Kong; 8 Department of Epidemiology, Biostatistics, Population Studies, and Health Promotion, Faculty of Public Health, Universitas Airlangga, Surabaya, 60115 Indonesia; 9 Department of Nutrition, Faculty of Medicine and Health Sciences, Universiti Putra Malaysia, 43400 Serdang, Selangor Malaysia; 10 Department of Turkish and Social Sciences Education, Faculty of Education, Anadolu University, Eskişehir, Turkey; 11 Department of Family Medicine, Cardinal Tien Hospital, 362 Zhongzheng Rd., New Taipei, 231009 Taiwan; 12 Department of Laboratory Medicine, National Taiwan University Hospital, 7 Chung Shan S. Rd., Taipei, 100225 Taiwan; 13 Division of Hematology and Internal Medicine, National Taiwan University Hospital, 7 Chung Shan S. Rd., Taipei, 100225 Taiwan; 14 Graduate Institute of Clinical Medicine, National Taiwan University, 1, Sec. 4, Roosevelt Rd., Taipei, 106319 Taiwan; 15 Research Unit for General Practice, Department of Public Health, University of Southern Denmark, Odense, Denmark; 16 DRIVEN - Danish Centre for Motivational and Behaviour Science, Department of Sports Science and Clinical Biomechanics, University of Southern Denmark, Odense, Denmark; 17 Research Unit for General Practice, Aarhus, Denmark; 18 School of Social Sciences, Monash University, 20 Chancellors Walk, Clayton, VIC 3800 Australia; 19 Psychology Department, Nottingham Trent University, 50 Shakespeare St., Nottingham, NG1 4FQ UK; 20 Department of Nursing, School of Health and Welfare, Jönköping University, Hälsohögskolan, Jönköping, 55318 Sweden; 21 Institute of Allied Health Sciences, College of Medicine, National Cheng Kung University, No. 1 University Rd., East District, Tainan, 701401 Taiwan; 22 Biostatistics Consulting Center, National Cheng Kung University Hospital, College of Medicine, National Cheng Kung University, 1 University Rd., Tainan, 701401 Taiwan; 23 Department of Public Health, College of Medicine, National Cheng Kung University, 1 University Rd., Tainan, 701401 Taiwan; 24 Department of Occupational Therapy, College of Medicine, National Cheng Kung University, 1 University Rd., Tainan, 701401 Taiwan; 25 School of Nursing, College of Nursing, Kaohsiung Medical University, Kaohsiung, 807378 Taiwan

**Keywords:** Weight stigma, Culture, Item-response theory, Asian countries, Weight bias

## Abstract

**Background:**

The Weight Stigma Exposure Inventory (WeSEI) is a newly developed instrument designed to assess weight stigma exposure across both interpersonal and non-interpersonal contexts. While prior studies have supported its use in individual regions, its cross-cultural applicability has not been comprehensively evaluated.

**Objective:**

The present study examined the psychometric properties and cross-cultural measurement equivalence of the WeSEI across six culturally diverse jurisdictional regions in Asia (i.e., Taiwan, China, Hong Kong, Indonesia, Türkiye, and Malaysia).

**Methods:**

A total of 7,787 participants completed the 35-item WeSEI via various online platforms. The WeSEI assesses weight stigma exposure across seven domains: social media, traditional media, television/movies, parents/siblings, friends/peers, significant others, and strangers. Rasch analysis was conducted to evaluate item fit, rating scale functioning, person separation reliability, and unidimensionality for each domain. Differential item functioning (DIF) was assessed across sex, weight status, and jurisdictional region.

**Results:**

The WeSEI demonstrated strong internal consistency, acceptable item fit, and unidimensionality across all domains, with only two items showing misfit (i.e., Friends/Peers_2 and Significant Other_2). Person separation indices exceeded 2.0 for each domain, confirming the scale’s ability to distinguish individuals with varying levels of stigma exposure. Minimal DIF was observed by sex and weight status, supporting measurement equivalence across these groups. However, 19 out of 35 items showed significant jurisdictional region-level DIF, particularly those related to slim-normative attractiveness and family-based stigma. Malaysian participants consistently endorsed these items more than Chinese participants, suggesting cultural variation in the exposure of weight stigma.

**Conclusion:**

The WeSEI is a psychometrically sound and culturally responsive tool for assessing weight stigma exposure across diverse populations in Asia. Cultural adaptation is recommended for cross-national comparisons.

## Abstract (plain language summary)

People who live in larger bodies often face unfair treatment because of their weight. This is called weight stigma. It can occur in many areas of life, such as on social media, on television and in movies, and in everyday interactions with family, friends, or strangers. To better understand how often and in what ways people experience weight stigma, researchers need good instruments that can assess these experiences clearly and fairly across different groups and cultures.

In this study, we examined the Weight Stigma Exposure Inventory (WeSEI). The WeSEI asks people about how often they encounter weight stigma in seven areas of life: social media, traditional media, TV and movies, parents or siblings, friends or peers, romantic partners, and strangers. We collected responses from 7,787 adults living in six regions in Asia: Taiwan, China, Hong Kong, Indonesia, Türkiye, and Malaysia. We then tested whether the WeSEI works well and assesses stigma exposure in a reliable way.

Our results showed that the WeSEI is a strong and consistent instrument for assessing weight stigma across all seven areas. Almost all items behaved as expected, and the instrument distinguished between people who experienced more stigma and those who experienced less. The WeSEI also worked similarly for people of different sexes and body weight categories, which suggests it is fair to these groups. However, some items worked differently across regions, especially those that focused on cultural ideas about being slim and on family-related weight comments. For example, individuals from Malaysia tended to report more agreement with these items than individuals from China, which may reflect differences in how weight stigma appears in daily life in these places.

Overall, the WeSEI appears to be a useful and culturally responsive instrument for studying weight stigma in diverse Asian populations. At the same time, the findings highlight that culture shapes how weight stigma is experienced and reported. Researchers and clinicians who use the WeSEI in cross-country studies should take these cultural differences into account and consider adapting specific items so that comparisons across regions are fair and accurate.

## Introduction

Weight stigma, defined as the social devaluation of individuals based on body weight, is a pervasive phenomenon [1]. It poses significant threats to both psychological and physical health [2]. Accumulating evidence has consistently associated weight stigma with a wide range of adverse outcomes, including body dissatisfaction [3], disordered eating [4, 5], depression and anxiety [6], low self-esteem [7], and the avoidance of healthcare [8, 9]. Importantly, weight stigma is not only experienced through overt acts of discrimination but also manifests in subtle and normalized sociocultural messaging, such as the idealization of thinness in mass media and daily interpersonal interactions [10]. While much of the existing literature has focused on Western populations, emerging studies in Asia suggested that weight stigma is also prevalent in non-Western societies, where it may be shaped by distinct cultural norms and values, and social dynamics [11]. This highlights the critical need for measurement instruments that are psychometrically robust and culturally sensitive to diverse contexts in the global literature.

Observed weight stigma (or weight stigma exposure), defined as the stigma individuals’ witness in their environment rather than direct experience, plays a critical role in shaping attitudes and health outcomes [12]. Exposure to weight-based teasing, biased media portrayals, and/or discriminatory behaviors toward others can normalize weight prejudice and contribute to the internalization of weight stigma, even among individuals who are not personally targeted [10, 13]. Importantly, one study found that thin-ideal internalization and internalized weight stigma have been shown to be independent predictors of body dissatisfaction, regardless of weight status [14].

Such vicarious exposure has been associated with adverse outcomes, including body dissatisfaction [15], engagement in unhealthy weight-control behaviors, elevated stress, and social withdrawal [16, 17]. These effects may vary across populations depending on cultural attitudes toward body size, prevailing social norms, and the degree of stigma internalization [18]. Therefore, understanding these mechanisms is essential for developing culturally sensitive interventions that adequately capture the breadth of stigma exposure across diverse populations.

In response to the need for a more comprehensive and context-sensitive tool, the Weight Stigma Exposure Inventory (WeSEI)was developed by scholars from four different countries (i.e., Taiwan, Thailand, United States, and Australia), to assess the frequency of observed weight-based stigma across multiple sources [19]. The WeSEI comprises 35 items across seven domains: *Social Media*, *Traditional Media*, *Television/Movies*, *Parents/Siblings*, *Friends/Peers*, *Significant Others*, and *Strangers*. This multidimensional structure allows for assessment of stigma exposure across both interpersonal and non-interpersonal contexts. Initial validations of the WeSEI among national samples from Malaysia [20], Hong Kong [21], Türkiye [22], Taiwan and China [19], Indonesia [23], Thailand [24], have shown promising psychometric properties, including satisfactory internal consistency, construct validity, concurrent validity, and measurement invariance across subgroups.

Although these early validations provide foundational evidence for the WeSEI’s reliability, the cross-national measurement equivalence of the WeSEI has not yet been rigorously evaluated. Establishing measurement equivalence is essential to ensure that observed differences in stigma exposure across cultural groups reflect true differences in experience, rather than artifacts of culturally biased measurement. Understanding whether the WeSEI functions equivalently across cultural groups is essential for international research and global health surveillance, where culturally appropriate assessment instruments are crucial for developing equitable policies and interventions.

Cultural differences in body image ideals, interpersonal communication norms, interpretations of interpersonal mistreatment and perceptions of stigma may influence how individuals interpret and respond to items on the WeSEI [18]. Without evaluation of item-level invariance, cross-cultural comparisons may lead to misleading or invalid conclusions about the nature and extent of observed weight stigma among different populations.

The specific objectives of the study were to: (i) assess the item fit of the 35 WeSEI items using Rasch Infit and Outfit mean square (MnSq) statistics, (ii) evaluate the functioning of the rating scale structure and unidimensionality, and ensure orderly response category thresholds across the five-point Likert scale, (iii) investigate differential item functioning (DIF) by sex, overweight status, and jurisdictional region to identify potential measurement bias across subgroups, and (iv) determine the overall construct validity and potential areas requiring cultural adaptation to enhance measurement equivalence of the WeSEI.

## Methods

### Study design, study procedures and participants

The present study utilized secondary data analysis by combining datasets previously collected to evaluate the psychometric properties of the WeSEI in six jurisdictional regions: Taiwan, China, Hong Kong, Indonesia, Türkiye, and Malaysia. Data were collected between September 2023 and November 2024 with the earliest data collected from Taiwan and China, and the most recent data collected from Türkiye. Ethical approval for the original data collection was obtained from the relevant research ethics committees or institutional review boards in each jurisdiction: National Cheng Kung University Human Research Ethics Committee (Approval No.: NCKU HREC-E-111–563-2) for Taiwan data; Institute Review Board of Jiangxi Psychological Consultant Association (JXSXL-2023-SE0906) for China data; Chinese University of Hong Kong Ethics Committee (Ethics Number: SBRE-23-0685) for Hong Kong data; Health Research Ethics Committee in Universitas Airlangga (Number: 188/EA/KEPK/2024) for Indonesia data; Anadolu University Social and Human Sciences Ethics Committee (No. 696904) for Türkiye data; and Ethics Committee for Research Involving Human Subjects in University Putra Malaysia (Reference Number: JKEUPM-2023-1324) for Malaysia data.

All non-English versions of the WeSEI used in this secondary analysis were developed in the primary validation studies following international guidelines for translation and cross-cultural adaptation of self-report instruments [25]. Each language version underwent forward translation by bilingual experts, back translation into English, and was then validated by an expert panel to ensure conceptual equivalence. The present study used validated versions of the WeSEI in Traditional Chinese Mandarin for Taiwan, Simplified Chinese Mandarin for mainland China, Cantonese Chinese for Hong Kong, Bahasa Indonesian for Indonesia, Malay for Malaysia, and Turkish for Türkiye.

Further details on data collection and participant eligibility for each jurisdictional region are described elsewhere (Taiwan and China [19], Hong Kong [21], Türkiye [22], and Malaysia [20], and Indonesia [23]). The primary inclusion criterion for this multi-jurisdictional region study was being aged 18 years or older. In addition, all participants completed the WeSEI via online questionnaire platforms, hosted on either *SurveyMonkey* (Taiwan, Indonesia, and Türkiye), *SoJump* (China), *Qualtrics* (Hong Kong), or *Google Form* (Malaysia). In order to ensure data quality, IP tracking (for data collection using commercial online survey platforms such as *SurveyMonkey*) and/or attention check items were used. Local research teams distributed the WeSEI survey links through their academic or professional networks, and social media platforms to recruit participants. Interested individuals who clicked on the survey link were first presented with an informed consent page that described the study purpose, procedures, and confidentiality. Those who confirmed that they met the inclusion criteria and provided electronic informed consent then completed the online survey, which included the WeSEI and demographic items. All participants who started the survey answered all the items.

### Measures

The Weight Stigma Exposure Inventory (WeSEI) is designed to assess the frequency of experiencing weight-based stigma across different media sources and interpersonal contexts. The inventory consists of 35 items, systematically organized into seven distinct domains, each comprising five items. These domains represent different sources of weight stigma exposure: *Social Media* (Domain 1), *Traditional Media* (Domain 2), *Television/Movies* (Domain 3), *Parents/Siblings* (Domain 4), *Friends/Peers* (Domain 5), *Significant Others* (Domain 6), and *Strangers* (Domain 7). Participants self-rate each item using a five-point Likert scale, where 1 corresponds to “Never,” 2 to “Seldom,” 3 to “Sometimes,” 4 to “Often,” and 5 to “Almost always.” Domain scores are calculated by summing the five items within each domain, yielding a possible range from 5 to 25. Higher scores indicate more frequent exposure to weight stigma from the specific corresponding source. The WeSEI enables both domain-specific and overall assessments of observed weight stigma.

Participants reported their age in years and sex, as well as their self-reported height (in centimeters) and weight (in kilograms). Body mass index (BMI) was calculated as weight in kilograms divided by height in meters squared (kg/m²).

### Data analysis

Participants’ demographic characteristics were summarized using descriptive statistics, including means and standard deviations (SDs) for continuous variables, and frequencies and percentages for categorical variables. Chi-square tests and one-way analysis of variance (ANOVA) were conducted to examine demographic differences and exposure levels across the 35 WeSEI items, using IBM SPSS Statistics (Version 29) analytics software [26].

To rigorously evaluate measurement equivalence, the present study applied Rasch modeling as a theory-driven framework to evaluate the cross-cultural psychometric properties of the WeSEI. Rasch analysis provides sample-independent item calibrations and allows for the evaluation of item fit, unidimensionality, rating scale functioning, and DIF across subgroups [27]. Rasch analysis is particularly well-suited for validating instruments such as the WeSEI, which rely on Likert-scale responses to assess latent constructs across culturally heterogeneous samples. It enables researchers to identify items that may function differently across groups and determine whether such differences reflect true variance in stigma exposure or cultural bias in item interpretation. Applying Rasch modeling ensures that inferences drawn from cross-national comparisons are grounded in measurement equivalence. Therefore, to address these gaps, the present study assessed the psychometric properties and cross-cultural validity of the WeSEI using Rasch analysis across six jurisdictional regions, comprising Taiwan, China, Hong Kong, Indonesia, Türkiye, and Malaysia.

Rasch analysis was performed with the software Facets (Version 4.4.0) and Winsteps (Version 5.10.1) to evaluate the psychometric properties of the WeSEI. Consistent with prior studies that conducted domain specific analyses [19, 20, 22], the seven domains of the WeSEI (i.e., *Social Media*, *Traditional Media*, *Television/Movies*, *Parents/Siblings*, *Friends/Peers*, *Significant Others*, and *Strangers*) were analyzed separately to evaluate their unidimensionality and measurement performance. Given the polytomous nature of the five-point Likert response format in WeSEI, the partial credit model was employed to estimate item calibrations and response category thresholds within each domain. This approach allows for flexible modeling of item-level rating scale structures and is appropriate for instruments where different items may exhibit varying step difficulties [28].

Rating scale functioning was assessed following Linacre’s criteria [29], ensuring that: (i) response categories increased monotonically in average measures across the five-point Likert scale in the WeSEI, (ii) each item had at least 10 responses per rating category, (iii) the person separation index exceeded 2.0, indicating that the WeSEI scale could distinguish at least three distinct levels of perceived weight stigma exposure, and (iv) item fit statistics met acceptable thresholds (Infit MnSq < 1.5 and Z_std_ < 2.0), indicating good model-data fit [30]. To evaluate dimensionality, principal components analysis (PCA) of the residuals was conducted for each subdomain. Unidimensionality is supported when the eigenvalue of the first contrast is less than 3.0, indicating minimal unexplained variance in the residuals [30].

Identifying DIF is critical to ensure that the WeSEI items assess weight stigma exposure comparably across diverse populations [31]. Therefore, DIF was examined to determine whether items functioned equivalently across key subgroups [32]. More specifically, DIF across sex (male vs. female), BMI group (overweight vs. non-overweight), and jurisdictional region (Taiwan, Hong Kong, China, Malaysia, Indonesia, and Türkiye) were tested. A BMI cut-off of ≥ 23 kg/m² was categorized as overweight for Asian participants [33, 34]. The Rasch-Welch *t*-test was used to detect statistically significant DIF (*p* < .05), and meaningful DIF was defined by an absolute contrast greater than 0.64 logits [35], indicating potential item bias and measurement non-equivalence across subgroups.

## Results

### Demographics

The demographic characteristics of the study sample (*N* = 7787) were examined across six jurisdictional regions: Taiwan, Hong Kong, China, Indonesia, Türkiye, and Malaysia. Participant age differed significantly by jurisdictional region (F = 1257, *p* < .001). The highest mean age was observed among participants from Taiwan (M = 28.82 years, SD = 6.1) and the youngest participants were from China (M = 18.93 years, SD = 1.5). Sex distribution also varied significantly across jurisdictional regions (χ² = 186.3, *p* < .001), with female participants comprising the majority in all samples. The highest proportion of female participants was in Indonesia (83.5%), and the lowest in China (63.6%).

The mean BMI also varied significantly between jurisdictional regions (F = 96.8, *p* < .001). China had participants with the highest average BMI (M = 25.00, SD = 8.5), while Hong Kong had the lowest (M = 21.10, SD = 3.5). Using the Asia-Pacific BMI classification where BMI ≥ 23 kg/m² is considered overweight, significant differences in overweight status prevalence were found across jurisdictional regions (χ² = 189.2, *p* < .001). The proportion of participants classified as overweight was highest in Taiwan (45.5%) and lowest in Hong Kong (20.9%). Overweight prevalence in the other jurisdictional regions was as follows: Türkiye (42.2%), China (39.8%), Indonesia (31.1%), and Malaysia (30.4%). Other details can be found in Table 1.

**Table 1:** Demographics (*N* = 7787)

Variables	Taiwan (*n* = 887)	China (*n* = 3135)	Hong Kong (*n* = 1008)	Indonesia (*n* = 1303)	Türkiye (*n* = 410)	Malaysia (*n* = 1044)	F or χ^2^	*p*
Age [M (SD)]	28.82 (6.1)	18.93 (1.5)	23.09 (5.3)	20.24 (1.3)	22.3 (4.6)	21.27 (2.5)	1257	< 0.001**
Gender [n (%)]								
Male	319 (36.0)	1142 (36.4)	314 (31.2)	215 (16.5)	107 (26.1)	317 (30.4)	186.3	< 0.001**
Female	568 (64.0)	1993 (63.6)	694 (68.8)	1088 (83.5)	303 (73.9)	727 (69.6)		
BMI [M (S.D.)]	22.80 (4.0)	25.00 (8.5)	21.10 (3.5)	21.79 (4.3)	22.88 (4.2)	21.92 (4.5)	96.8	< 0.001**
BMI Status [n (%)]								
Overweight (> 23)	404 (45.5)	1248 (39.8)	211 (20.9)	405 (31.1)	173 (42.2)	318 (30.5)	189.2	< 0.001**
Non-overweight (≤ 23)	483 (54.5)	1887 (60.2)	797 (89.1)	898 (68.9)	237 (57.8)	726 (69.5)		

N = Number; M = Mean; SD = Standard Deviation; BMI = Body Mass Index. ** *p* < .001

Table 2 presents the average item scores on the 35 WeSEI items across six jurisdictional regions. Significant cross-jurisdictional region differences were found for all 35 items (*p* < .001), indicating substantial variability in weight stigma exposure. Overall, participants from Malaysia and Indonesia reported the highest levels of weight stigma exposure across most domains. Malaysia consistently showed the highest summed scores in *Social Media*, *Television/Movies*, *Friends/Peers*, and *Parents/Siblings*-related stigma sources, while Indonesia reported the highest scores in the *Traditional Media* domain. In contrast, China demonstrated the lowest scores across all domains, indicating comparatively lower reported exposure to weight stigma.

**Table 2:** WeSEI item average scores across six jurisdictional regions (*N* = 7787)

Variables	Taiwan (*n* = 887)	China (*n* = 3135)	Hong Kong (*n* = 1008)	Indonesia (*n* = 1303)	Türkiye (*n* = 410)	Malaysia (*n* = 1044)	F	*p*
Social_1	2.37	2.08	2.37	2.87	2.64	3.01	152.1	< 0.001**
Social_2	3.09	2.46	3.06	3.69	3.25	3.78	293.0	< 0.001**
Social_3	2.74	2.44	2.81	3.43	3.17	3.45	205.6	< 0.001**
Social_4	2.71	2.24	2.74	2.97	3.18	3.25	169.8	< 0.001**
Social_5	2.42	2.12	2.39	2.84	3.01	3.07	155.0	< 0.001**
Tradition_1	2.28	1.95	2.14	2.86	2.51	2.37	159.2	< 0.001**
Tradition_2	2.76	2.09	2.46	3.39	2.92	2.99	295.4	< 0.001**
Tradition_3	2.42	2.05	2.19	3.05	2.75	2.61	185.4	< 0.001**
Tradition_4	2.39	1.98	2.13	2.84	2.69	2.48	148.5	< 0.001**
Tradition_5	2.25	1.95	2.01	2.67	2.56	2.34	107.3	< 0.001**
TV_1	2.63	2.25	2.53	2.77	2.86	2.97	92.6	< 0.001**
TV_2	3.17	2.52	3.07	3.53	3.56	3.63	223.5	< 0.001**
TV_3	2.79	2.36	2.70	2.90	3.04	3.03	285.6	< 0.001**
TV_4	2.87	2.42	2.76	2.97	2.90	3.21	92.0	< 0.001**
TV_5	2.79	2.42	2.67	2.96	2.99	3.24	92.7	< 0.001**
Parent_1	2.12	1.86	2.08	2.31	2.11	2.35	47.7	< 0.001**
Parent_2	2.46	1.94	2.32	2.60	2.31	2.78	104.6	< 0.001**
Parent_3	2.21	1.87	2.15	2.16	1.66	1.98	34.0	< 0.001**
Parent_4	1.96	1.79	1.96	1.93	1.79	2.15	17.2	< 0.001**
Parent_5	1.64	1.64	1.62	1.72	1.46	1.66	4.9	< 0.001**
Friends_1	2.16	1.85	2.22	2.06	2.16	2.31	42.8	< 0.001**
Friends_2	2.71	2.04	2.62	2.74	2.59	3.01	137.6	< 0.001**
Friends_3	2.15	1.83	2.20	2.01	1.91	2.03	29.7	< 0.001**
Friends_4	2.04	1.74	2.02	1.94	1.99	2.16	33.1	< 0.001**
Friends_5	1.81	1.67	1.78	1.78	1.72	1.80	5.9	< 0.001**
Other_1	1.91	1.73	1.84	1.63	1.74	1.78	10.6	< 0.001**
Other_2	2.34	1.85	2.15	1.92	2.10	2.15	36.6	< 0.001**
Other_3	2.05	1.77	1.94	1.76	1.81	1.76	14.6	< 0.001**
Other_4	1.83	1.68	1.73	1.56	1.63	1.69	9.8	< 0.001**
Other_5	1.59	1.64	1.59	1.51	1.42	1.52	7.8	< 0.001**
Stranger_1	2.57	2.19	2.46	2.51	2.52	2.91	61.9	< 0.001**
Stranger_2	2.93	2.32	2.70	3.03	2.94	3.31	128.7	< 0.001**
Stranger_3	2.68	2.22	2.55	2.72	2.59	2.87	73.0	< 0.001**
Stranger_4	2.44	2.17	2.37	2.61	2.62	2.88	68.6	< 0.001**
Stranger_5	2.26	2.05	2.18	2.50	2.45	2.71	64.8	< 0.001**

WeSEI = Weight Stigma Exposure Inventory. ** *p* < .001

### Rasch item fit and rating scale functioning

Rasch analyses were conducted separately for each of the seven WeSEI domains. The five-point Likert rating scale functioned appropriately across all domains. More specifically, average measures increased monotonically across response categories, and all items had at least 10 responses per rating category, meeting Linacre’s criteria for optimal rating scale functioning [29]. Person separation indices exceeded the recommended threshold of 2.0 in all domains, indicating that the WeSEI reliably distinguished at least three distinct levels of weight stigma exposure among participants. The person separation indices for the seven domains were as follows: *Social Media* (2.86), *Traditional Media* (3.35), *TV/Movies* (3.61), *Parents/Siblings* (2.18), *Friends/Peers* (2.30), *Significant Others* (2.02), and *Strangers* (3.22). Corresponding person reliability estimates ranged from 0.93 to 0.99, reflecting high internal consistency and stable measurement of the latent trait.

Table 3 presents the item calibrations, standard errors, and fit statistics for all 35 WeSEI items. Each domain was analyzed separately. These calibrations formed a hierarchy that reflects the relative difficulty of endorsing each item along the weight stigma exposure continuum. Across all seven domains, Item 2 (e.g., *“I have observed that people consider being slim to be more attractive than being overweight”*) consistently emerged as the easiest to endorse, with calibration values ranging from − 1.67 to − 0.96. This suggested that such stigmatizing beliefs were the most observed or reported across different social contexts. In contrast, the most difficult items to endorse, indicated by the highest calibration values, varied by domain. Item 1 was the most difficult in the *Social Media* domain (*“I have observed negative statements about weight on social media”*) and the *TV/Movies* domain (*“The TV series or movies I watched portrayed individuals who are overweight in a negative way”*), with calibration values reaching up to 0.98. In the remaining five domains, including *Traditional Media*, *Parents/Siblings*, *Friends/Peers*, *Significant Others*, and *Strangers*, Item 5 [using Traditional Media Item 5 as an example, “*I have seen negative behaviors towards people who are overweight (e.g.*,* I kick those with a fat ass) from traditional media (e.g.*,* television*,* newspaper*,* and magazines)*”] consistently showed the highest calibration values, up to 1.21. These items reflected direct mistreatment of individuals who are overweight by individuals in those contexts.

**Table 3:** Rasch analyses of the 35 WeSEI items

Item	Measure	SE	Infit		Outfit		Average Rating Measure^a^				
			MnSq	Z_std_	MnSq	Zstd	1	2	3	4	5
Social_1	0.81	0.02	1.21	9.90	1.23	9.90	-4.32	-1.45	0.24	1.94	4.83
Social_2	− 0.96	0.02	1.13	7.10	1.12	6.30	-5.63	-2.32	− 0.71	0.72	2.76
Social_3	− 0.53	0.02	0.79	-9.90	0.79	-9.90	-5.71	-2.28	− 0.46	1.26	3.72
Social_4	0.06	0.02	0.83	-9.90	0.83	-9.90	-5.15	-1.83	− 0.17	1.64	4.31
Social_5	0.60	0.02	1.01	0.68	1.01	0.33	-4.5	-1.53	0.12	1.90	4.91
Tradition_1	0.49	0.03	1.02	1.06	0.98	− 0.88	-7.49	-2.86	0.22	2.79	6.67
Tradition_2	-1.20	0.02	1.38	9.90	1.43	9.90	-8.22	-3.59	− 0.78	1.25	3.99
Tradition_3	− 0.20	0.03	0.77	-9.90	0.74	-9.90	-7.91	-3.25	− 0.13	2.29	5.76
Tradition_4	0.21	0.03	0.77	-9.90	0.73	-9.90	-7.62	-2.98	0.08	2.68	6.37
Tradition_5	0.70	0.03	0.97	-1.65	0.95	-2.53	-7.07	-2.70	0.32	2.93	6.64
TV_1	0.98	0.02	1.20	9.90	1.21	9.90	-6.59	-2.33	0.53	3.18	7.29
TV_2	-1.23	0.02	1.40	9.90	1.43	9.90	-7.71	-3.36	− 0.60	1.36	4.19
TV_3	0.25	0.02	0.82	-9.90	0.80	-9.90	-7.09	-2.68	0.19	2.78	6.54
TV_4	− 0.02	0.02	0.70	-9.90	0.68	-9.90	-7.32	-2.93	− 0.01	2.61	6.50
TV_5	0.03	0.02	0.80	-9.90	0.77	-9.90	-7.23	-2.85	0.06	2.61	6.36
Parent_1	− 0.29	0.02	0.85	-7.79	0.87	-7.11	-5.99	-2.41	− 0.53	0.95	3.67
Parent_2	-1.09	0.02	1.24	9.90	1.27	9.90	-6.30	-2.69	-1.02	0.07	2.45
Parent_3	− 0.10	0.02	0.87	-6.69	0.88	-6.49	-5.75	-2.20	− 0.40	0.93	3.63
Parent_4	0.28	0.02	0.80	-9.90	0.83	-8.48	-5.46	-2.02	− 0.25	1.38	4.28
Parent_5	1.21	0.02	1.22	9.90	1.13	3.91	-4.66	-1.61	0.18	1.85	5.16
Friends_1	0.01	0.02	0.78	-9.90	0.78	-9.90	-6.37	-2.40	− 0.35	1.28	4.34
Friends_2	-1.67	0.02	1.74^+^	9.90^+^	1.82	9.90	-7.03	-2.89	-1.22	− 0.40	1.13
Friends_3	0.19	0.02	0.76	-9.90	0.76	-9.90	-6.07	-2.21	− 0.25	1.31	4.36
Friends_4	0.44	0.02	0.73	-9.90	0.74	-9.90	-5.81	-2.07	− 0.15	1.59	4.73
Friends_5	1.03	0.02	0.95	-2.49	0.92	-3.90	-5.17	-1.78	0.11	2.11	5.25
Other_1	0.15	0.03	0.79	-9.90	0.79	-9.90	-6.66	-2.38	− 0.19	1.44	4.51
Other_2	-1.22	0.03	1.56^+^	9.90^+^	1.72	9.90	-7.09	-2.76	− 0.94	− 0.22	1.87
Other_3	− 0.31	0.03	0.83	-7.76	0.84	-7.45	-6.78	-2.50	− 0.34	0.81	3.47
Other_4	0.45	0.03	0.75	-9.90	0.78	-9.90	-6.39	-2.17	0.03	1.61	4.77
Other_5	0.92	0.03	1.03	1.33	0.99	− 0.22	-5.97	-1.99	0.17	1.96	5.22
Stranger_1	0.23	0.02	0.98	-1.03	0.97	-1.78	-6.68	-2.79	− 0.15	2.33	6.11
Stranger_2	-1.09	0.02	1.27	9.90	1.28	9.90	-7.35	-3.21	− 0.67	1.10	3.63
Stranger_3	− 0.17	0.02	0.74	-9.90	0.72	-9.90	-7.11	-2.99	− 0.19	2.09	5.65
Stranger_4	0.23	0.02	0.80	-9.90	0.79	-9.90	-6.81	-2.67	− 0.06	2.45	6.11
Stranger_5	0.80	0.02	1.15	7.96	1.16	7.95	-6.88	-2.31	0.21	2.73	6.40

^a^The average measure is expected to increase with category value (Linacre, 2002). ^+^Infit statistics > 1.5 associated with Z_std_ > 2: item misfit. SE = standard errors; MnSq = Mean Square; Z_std_ = Standardized score

Overall, item-level fit statistics demonstrated strong alignment with the expectations of the Rasch model. However, two items showed notable misfit. *Friends/Peers_2* (*“My friends/peers think/believe that people with a slim shape are more attractive than those who are overweight”*) had an infit MnSq of 1.74 with a Z_std_ of 9.90, and *Significant Other_2* (*“My significant other finds people with a slim shape more attractive than those who are overweight”*) had an infit MnSq of 1.56 with a Z_std_ of 9.90. The unidimensionality of each domain was supported by PCA of the residuals. The eigenvalues of the first contrast were below the cutoff value of 3.0 for each domain (ranging from 1.71 to 1.94 across the seven domains).

### Differential item functioning (DIF)

Table 4 summarizes the results of DIF analysis across sex, overweight status, and jurisdictional region. For sex, 15 out of the 35 WeSEI items showed DIF contrasts favoring female participants (i.e., items easier to endorse by female participants). While most sex-related DIF contrasts were small, two items exceeded the meaningful DIF threshold of │0.64│, indicating potential measurement bias. More specifically, *Friends/Peers_2* (*“My friends/peers think/believe that individuals with a slim shape are more attractive than those who are overweight”*) showed a contrast of 0.79 (favoring females), and *Parents/Siblings_5* (*“My parents and siblings have treated individuals who are overweight badly”*) showed a contrast of -0.69 (favoring males). For overweight status, DIF contrasts were generally small and fell well below the │0.64│ threshold and the effects were negligible.

**Table 4:** DIF contrast for the 35 WeSEI items

Item	DIF Contrast					
	Gender^a^	Overweight Status^b^	Country^c^			
			Min	Country Pair	Max	Country Pair
Social_1	− 0.09	− 0.12	0.00	Taiwan vs. Malaysia	− 0.52	China vs. Turkey
Social_2	0.46	− 0.02	− 0.11	Taiwan vs. Hong Kong	− 0.90	Indonesia vs. Turkey
Social_3	0.22	0.08	0.06	Türkiye vs. Malaysia	0.51	Taiwan vs. Indonesia
Social_4	− 0.30	0.07	0.05	Taiwan vs. Hong Kong	0.85	Indonesia vs. Turkey
Social_5	− 0.29	− 0.02	0.01	Taiwan vs. Malaysia	0.68	Hong Kong vs. Turkey
Tradition_1	− 0.05	0.02	− 0.07	Türkiye vs. Malaysia	− 0.61	Hong Kong vs. Malaysia
Tradition_2	0.29	0.11	0.18	Indonesia vs. Malaysia	1.33	China vs. Malaysia
Tradition_3	0.20	0.06	0.01	Taiwan vs. Hong Kong	0.31	Taiwan vs. Indonesia
Tradition_4	− 0.13	− 0.03	0.01	Taiwan vs. China	0.47	Indonesia vs. Turkey
Tradition_5	− 0.34	− 0.17	− 0.06	Hong Kong vs. Malaysia	− 0.83	China vs. Indonesia
TV_1	− 0.42	− 0.08	− 0.01	Taiwan vs. Hong Kong	− 0.30	China vs. Indonesia
TV_2	0.23	0.05	0.06	Taiwan vs. Hong Kong	1.33	China vs. Indonesia
TV_3	− 0.02	− 0.04	− 0.03	China vs. Hong Kong	− 0.63	Turkey vs. Malaysia
TV_4	− 0.02	0.01	− 0.03	Taiwan vs. Hong Kong	− 0.79	China vs. Turkey
TV_5	0.22	0.06	0.02	Hong Kong vs. Türkiye	− 0.46	China vs. Hong Kong
Parent_1	0.21	0.00	0.04	Taiwan vs. China	0.81	Taiwan vs. Turkey
Parent_2	0.40	0.00	0.22	Taiwan vs. Indonesia	1.33	China vs. Malaysia
Parent_3	0.06	0.13	0.02	Taiwan vs. Hong Kong	-1.31	Hong Kong vs. Turkey
Parent_4	− 0.11	− 0.08	0.02	China vs. Malaysia	− 0.61	China vs. Indonesia
Parent_5	− 0.69	− 0.06	− 0.02	Taiwan vs. Hong Kong	-1.13	China vs. Malaysia
Friends_1	0.08	0.04	− 0.02	Hong Kong vs. Malaysia	0.46	Indonesia vs. Turkey
Friends_2	0.79	0.18	0.14	Taiwan vs. Turkey	1.37	China vs. Malaysia
Friends_3	− 0.04	0.07	0.09	Taiwan vs. China	− 0.91	Hong Kong vs. Malaysia
Friends_4	− 0.46	− 0.14	− 0.03	Türkiye vs. Malaysia	0.29	Indonesia vs. Turkey
Friends_5	− 0.50	-18	− 0.01	Hong Kong vs. Türkiye	− 0.91	China vs. Malaysia
Other_1	− 0.06	− 0.10	− 0.01	China vs. Turkey	0.26	Indonesia vs. Malaysia
Other_2	0.40	0.26	− 0.01	Hong Kong vs. Indonesia	1.31	China vs. Malaysia
Other_3	0.19	0.07	0.02	Taiwan vs. Hong Kong	− 0.60	Indonesia vs. Malaysia
Other_4	− 0.18	− 0.10	− 0.05	Taiwan vs. Malaysia	− 0.42	China vs. Indonesia
Other_5	− 0.43	− 0.18	− 0.21	Hong Kong vs. Malaysia	-1.42	China vs. Turkey
Stranger_1	− 0.07	− 0.01	0.01	Taiwan vs. China	− 0.68	Hong Kong vs. Indonesia
Stranger_2	0.41	0.03	− 0.02	Taiwan vs. Indonesia	0.78	China vs. Malaysia
Stranger_3	0.02	0.01	0.08	Taiwan vs. China	− 0.65	Taiwan vs. Malaysia
Stranger_4	− 0.15	0.02	0.01	Indonesia vs. Malaysia	0.51	Taiwan vs. Turkey
Stranger_5	− 0.22	− 0.04	0.00	China vs. Indonesia	0.56	Taiwan vs. China

^a^ Negative values indicate that the probability of the item endorsement is easier for male participants than female participants

^b^ Negative values indicate that the probability of the item endorsement is easier for overweight participants than non-overweight participants

^c^ Negative values indicate that the probability of the item endorsement is easier for the first listed country than the second listed country

DIF = Differential Item Functioning

The analysis of DIF by jurisdictional region showed substantial cross-national variability in the endorsement of items on the WeSEI, with 19 out of 35 items showing meaningful DIF, defined as absolute contrast values exceeding 0.64 logits [29]. Notably, the same item across all the seven subdomains emphasizing slim-normative attractiveness were significant: *Social Media_2* (DIF = − 0.90), *Traditional Media_2* (DIF = 1.33), *TV/Movies_2* (DIF = 1.33), *Parents/Siblings_2* (DIF = 1.33), *Friends/Peers_2* (DIF = 1.37), *Significant Others_2* (DIF = 1.31), and *Strangers_2* (DIF = 0.78). Out of these seven items, five exhibited pronounced DIF when comparing China to Malaysia. Additionally, the *Parents/Siblings* domain exhibited the greatest cross-national variability, with four out of five items showing significant DIF.

## Discussion

The present study applied Rasch analysis across six culturally diverse populations in Asia (i.e., Taiwan, China, Hong Kong, Indonesia, Türkiye, and Malaysia) to assess the structural and construct validity of the WeSEI. The findings provided robust support for the psychometric performance of the WeSEI in these varied contexts. The present study has several notable strengths. It used a large, culturally diverse sample of young adults from six Asian jurisdictions and applied comprehensive Rasch modeling, including rating scale evaluation and DIF analyses which provided a rigorous examination of the WeSEI’s measurement properties across cultural contexts. Across all seven domains, the WeSEI demonstrated satisfactory internal consistency which was consistent with previous findings [20, 22]. Person separation indices exceeded the standard cutoff of 2.0, indicating that the WeSEI reliably distinguished the enrolled participants with varying levels of weight stigma exposure. PCA of the residuals also supported unidimensionality for the seven domains, and the five-point rating scale structure was confirmed.

Most items exhibited acceptable fit statistics to the Rasch model. However, two items (*Friends/Peers_2* and *Significant Others_2*) showed misfit, with infit MnSq statistics above the threshold of 1.5 associated with Z_std_ over 2. These patterns suggest that participants’ responses to these items were less consistent with participants’ overall levels of stigma exposure [36], possibly due to variability in interpretation. For example, for these two items specifically, some participants may have interpreted ‘friends/peers’ narrowly as a close-knit group, while others may have considered it more broadly to include casual acquaintances. This variability could lead to inconsistent responses even among participants with similar weight stigma exposure. Similarly, the term ‘significant other’ may be interpreted as a current romantic partner, a former partner, or even an aspirational figure, depending on personal contexts. Therefore, such ambiguity may have introduced measurement error and reduced item-level fit [30].

DIF analysis showed minimal item bias by sex and overweight status. Only two items exceeded the │0.64│ DIF threshold for sex: *Friends/Peers_2* (favoring females) and *Parents/Siblings_5* (favoring males). These findings indicate that the majority of WeSEI items function equivalently across sex. However, these two items may require further examination to assess potential sex-related response bias. In contrast, DIF analysis by overweight status showed negligible differences, supporting the measurement equivalence of the WeSEI between overweight and non-overweight participants. This result confirms that clinicians and researchers can use the WeSEI to compare results across BMI-defined groups in Asia. These findings were also consistent with previous validation studies that established measurement invariance by sex and weight status among Taiwanese and Chinese populations [19], as well as among the Malaysian population [20].

In contrast, jurisdictional region-level DIF was more substantial. A total of 19 out of 35 items exceeded the DIF threshold. In particular, items related to slim-normative attractiveness (specifically, Item 2 in each of the seven subdomains) showed significant DIF, with consistently higher endorsement in Malaysia compared to China. These findings aligned with prior validation studies among Malaysian populations, where slimness ideals are strongly reinforced through interpersonal relationships and media representations [20]. Additional research has shown that the body image is a major concern among Malaysian female college students [37]. Most recently, the Global Burden of Diseases, Injuries, and Risk Factors Study indicated that the prevalence of being overweight and obesity has substantially increased over time among Malaysians, especially females [38], and the same study projects the prevalence of being overweight to rise from 35.5% in 1990 to 72.8% in 2050 [38]. This may further intensify societal emphasis on slimness as an ideal, especially for females. In other words, Malaysians may be worried about the increased prevalence rate of being overweight, and therefore be more critical of weight. These findings emphasize the importance of interpreting DIF not as a flaw in the instrument, but as evidence of how the cultural context can shape the expression and perception of weight-related stigma.

The *Parents/Siblings* domain showed the highest cross-national DIF, with four of five items flagged, indicating that family-based weight stigma may be interpreted differently across cultural contexts. For example, *Parents/Siglings_2* (*“My parents and siblings think that people with a slim shape are more attractive than those who are overweight”*) showed the highest DIF contrast, with a value of 1.33 between China and Malaysia. This indicated that Malaysian participants were significantly more likely to endorse this item than Chinese participants, even when reporting similar levels of family-based weight stigma. One likely explanation is that in Malaysia, slimness ideals are widely reinforced through media, social norms, and public health messaging, making it more culturally acceptable for family members to express a preference for thinness directly [39]. As a result, Malaysian participants may view such statements as typical family attitudes and endorse them more readily. Comparatively, in Chinese cultural contexts, which are often characterized by indirectness, prioritizing harmony, and face-saving [40], these comments regarding slimness may be communicated more indirectly or softened through implicit language, leading to lower item endorsement despite similar experiences. Among all the family members, one study found that both mothers and sisters were the most important sources of pressure [41]. This finding highlights how cultural norms can influence item interpretation and participants’ perception. Therefore, awareness of the cultural adaptation and local validation is critical when using the WeSEI across different jurisdictional regions.

While cross-national DIF in the *Parents/Siblings* domain illustrated how cultural norms affect the way slimness ideals were expressed within families, the item calibration hierarchy provided additional insight into how those same cultural values influence individuals’ willingness to report mistreatment. The WeSEI calibration results further support the scale’s construct validity and offers meaningful understanding of how weight stigma was observed across different sources. Items reflecting implicit sociocultural beliefs, such as the idea that slimness is more attractive, were consistently the easiest to endorse across all seven domains. This suggests that weight-based appearance norms have become widely accepted and are rarely questioned in daily interactions and media exposure [42, 43].

In contrast, the items that were most difficult to endorse described direct mistreatment of individuals who were overweight. This difference in endorsement may be due to comments or judgments about appearance often being socially tolerated [44], whereas overt behaviors such as bullying or exclusion are viewed as more serious and less acceptable. In many Asian cultural contexts, especially those that value family harmony, respect for elders, and indirect communication, individuals may be reluctant to acknowledge or report mistreatment from close others [45]. For example, Dong et al. [46] found that Chinese elders often viewed mistreatment within the family as a private issue and were hesitant to disclose it.

Similarly, Azmi et al. [47] reported that Malaysian university students predominantly held collectivist values and preferred indirect ways of communicating, which influenced how they interpreted and responded to interpersonal experiences. These cultural norms may lead participants to minimize or reinterpret negative interactions with family or close relationships, making them less likely to view such experiences as stigmatizing. Consequently, participants in the present study may have underreported overt forms of mistreatment unless they were severe or persistent, even while acknowledging widespread slimness ideals.

### Limitations

Despite its strengths, the present study has several limitations. First, the data were cross-sectional and self-reported, which may be subject to recall and social desirability biases [48] and may also introduce biases related to internet access, digital literacy, and willingness to participate in web-based research. Consequently, participants may differ from nonparticipants in important ways, such as being younger, more educated, or more comfortable disclosing weight-related experiences online, which should be considered when generalizing these findings. Second, although the total sample size was large and geographically diverse, notable variations in age, sex distribution, and overweight prevalence across jurisdictional regions may limit the generalizability of findings to all cultural or socioeconomic subgroups within each jurisdictional region. These disparities suggested that specific demographic groups may be overrepresented, which could influence observed patterns of weight-related stigma exposure. For example, the sample was relatively young and predominantly female. Given that experiences and perceptions of weight stigma may change with age, it is unclear whether the observed patterns remain stable or shift as individuals grow older. Future research should aim to recruit more age-diverse samples and employ longitudinal designs to better capture how weight stigma experiences develop across the lifespan and within different cultural contexts.

Additionally, the sample included relatively few individuals with severe obesity, as indicated by the comparatively low mean BMI. Consequently, it remains uncertain whether experiences of weight stigma are comparable across countries among individuals with higher levels of obesity, and this potential variability should be considered when interpreting the findings. Moreover, the presence of jurisdictional region-level DIF indicated that specific items may require further cultural adaptation to ensure meaningful cross-cultural comparisons. Future studies should consider conducting cognitive interviews to explore how specific WeSEI items are interpreted in different cultural contexts. Lastly, the data collection method (i.e., using social media platforms to recruit participants) meant that the research team did not know how many people saw the recruitment advert or how many eligible participants were approached. Therefore, it was not possible to calculate a response rate.

### Implications for research and practice

The present study provides strong evidence that the WeSEI is a psychometrically valid and culturally responsive tool for assessing weight stigma exposure across diverse Asian populations. Its domain-specific structure allows researchers and clinicians to identify the primary sources of stigma within specific cultural contexts. The consistent endorsement of items reflecting slimness as an attractive ideal underscores the widespread normalization of weight-based appearance norms. Interventions should therefore prioritize addressing these implicit sociocultural values, particularly in countries such as Malaysia where such norms are strongly reinforced.

The significant cross-national DIF observed in family-related items highlights the importance of cultural adaptation and local validation when using the WeSEI. Practitioners should consider cultural differences in communication styles, family dynamics, and perceptions of mistreatment when designing culturally sensitive stigma-reduction strategies. Finally, the minimal DIF by sex and BMI status confirmed the WeSEI’s suitability for comparisons across demographic subgroups, supporting its use in both research and clinical applications aimed at promoting weight stigma awareness and reduction.

### Conclusion

The present study provided comprehensive psychometric validation of the WeSEI across six culturally diverse Asian populations using Rasch analysis. The WeSEI demonstrated strong construct validity, stable rating scale properties, high internal consistency, and unidimensionality in all seven domains. Minimal differential item functioning by sex and BMI status supports its appropriateness for subgroup comparisons. However, the presence of substantial jurisdictional region-level DIF underscores the importance of cultural adaptation when applying the WeSEI across international contexts. Overall, the study’s results support the use of the WeSEI as a reliable and culturally-sensitive tool for assessing weight stigma exposure.

## Data Availability

The data that support the findings of the present study are available from the corresponding author upon reasonable request.
